# Initial Training for Mental Health Peer Support Workers: Systematized Review and International Delphi Consultation

**DOI:** 10.2196/25528

**Published:** 2021-05-27

**Authors:** Ashleigh Charles, Rebecca Nixdorf, Nashwa Ibrahim, Lion Gai Meir, Richard S Mpango, Fileuka Ngakongwa, Hannah Nudds, Soumitra Pathare, Grace Ryan, Julie Repper, Heather Wharrad, Philip Wolf, Mike Slade, Candelaria Mahlke

**Affiliations:** 1 School of Health Sciences, Institute of Mental Health University of Nottingham Nottingham United Kingdom; 2 Department of Psychiatry University Medical Center Hamburg-Eppendorf Hamburg Germany; 3 Psychiatric and Mental Health Nursing Department, Faculty of Nursing Mansoura University Masoura Egypt; 4 Department of Social Work Ben Gurion University of the Negev Beer Sheva Israel; 5 Butabika National Referral Hospital Butabika Uganda; 6 School of Health Sciences Soroti University Soroti Uganda; 7 MRC/UVRI and LSHTM Uganda Research Unit Entebbe Uganda; 8 Ifakara Health Institute Dar es Salaam United Republic of Tanzania; 9 Department of Psychiatry and Mental Health Muhimbili University of Health and Allied Sciences Dar es Salaam United Republic of Tanzania; 10 Centre for Mental Health Law and Policy Indian Law Society Pune India; 11 Centre for Global Mental Health London School of Hygiene and Tropical Medicine London United Kingdom; 12 ImROC Nottinghamshire Healthcare NHS Foundation Trust Nottingham United Kingdom; 13 Faculty of Medicine and Health Sciences University of Nottingham Nottingham United Kingdom; 14 Department of Psychiatry II Ulm University II Ulm Germany

**Keywords:** peer support work, peer support worker training, Delphi consultation, mental health, mobile phone

## Abstract

**Background:**

Initial training is essential for the mental health peer support worker (PSW) role. Training needs to incorporate recent advances in digital peer support and the increase of peer support work roles internationally. There is a lack of evidence on training topics that are important for initial peer support work training and on which training topics can be provided on the internet.

**Objective:**

The objective of this study is to establish consensus levels about the content of initial training for mental health PSWs and the extent to which each identified topic can be delivered over the internet.

**Methods:**

A systematized review was conducted to identify a preliminary list of training topics from existing training manuals. Three rounds of Delphi consultation were then conducted to establish the importance and web-based deliverability of each topic. In round 1, participants were asked to rate the training topics for importance, and the topic list was refined. In rounds 2 and 3, participants were asked to rate each topic for importance and the extent to which they could be delivered over the internet.

**Results:**

The systematized review identified 32 training manuals from 14 countries: Argentina, Australia, Brazil, Canada, Chile, Germany, Ireland, the Netherlands, Norway, Scotland, Sweden, Uganda, the United Kingdom, and the United States. These were synthesized to develop a preliminary list of 18 topics. The Delphi consultation involved 110 participants (49 PSWs, 36 managers, and 25 researchers) from 21 countries (14 high-income, 5 middle-income, and 2 low-income countries). After the Delphi consultation (round 1: n=110; round 2: n=89; and round 3: n=82), 20 training topics (18 universal and 2 context-specific) were identified. There was a strong consensus about the importance of five topics: *lived experience as an asset*, *ethics*, *PSW well-being*, and *PSW role focus on recovery* and *communication*, with a moderate consensus for all other topics apart from the *knowledge of mental health*. There was no clear pattern of differences among PSW, manager, and researcher ratings of importance or between responses from participants in countries with different resource levels. All training topics were identified with a strong consensus as being deliverable through blended web-based and face-to-face training (rating 1) or fully deliverable on the internet with moderation (rating 2), with none identified as only deliverable through face-to-face teaching (rating 0) or deliverable fully on the web as a stand-alone course without moderation (rating 3).

**Conclusions:**

The 20 training topics identified can be recommended for inclusion in the curriculum of initial training programs for PSWs. Further research on web-based delivery of initial training is needed to understand the role of web-based moderation and whether web-based training better prepares recipients to deliver web-based peer support.

## Introduction

### Background

Peer support is rapidly developing into a central approach to support mental health recovery [1]. It has been defined as “a system of giving and receiving help founded on the key principles of respect, shared responsibility, and a mutual agreement of what is helpful” [2]. Formal peer support involves individuals with lived experience of mental health conditions and/or mental health services—variously called peer providers, peer specialists, peer support volunteers, or as peer support workers (PSWs)—who are engaged in a peer support capacity to support others’ recovery from mental health conditions [3].

There is strong empirical evidence for the positive benefits of implementing PSW roles. PSWs contribute to an increase in service user engagement, a sense of empowerment [4], improved social relationships [5], self-efficacy [6], hope [7], self-management [8], and positive clinical outcomes [9,10]. PSW roles are being implemented internationally, increasingly in lower middle-income countries such as India [11] and Uganda [12], where PSWs are engaged to address the mental health care gap [13].

Numerous PSW training programs exist [14,15]. These programs are designed to prepare individuals for the PSW role and sometimes also to meet the required competencies for accreditation. However, PSW roles and activities vary depending on their particular setting and context. For example, PSWs may work one-on-one or in a group setting with people who use mental health services, they may support individuals through service transitions, from hospital to the community, into employment, and/or to access local opportunities or resources. Training programs vary depending on the setting, local context, and resources.

Peer support has traditionally been provided as a face-to-face intervention. However, with the growth of digital mental health interventions changing the ways in which mental health care is delivered, peer support is increasingly being offered via digital technologies known as digital peer support. Digital peer support is defined as automated or live peer support services that can be delivered through multiple modalities [16], such as web-based peer support [17], smartphone-supported interventions [18,19], and web-based peer-to-peer networks [20]. A recent systematic review concluded that digital peer support appears to be both acceptable and feasible, with a strong potential for clinical effectiveness [16]. The use of web-based PSW approaches such as digital peer support is likely to increase in response to the COVID-19 global pandemic [21]. This new development means that PSW training may also be offered on the web, and some initial training topics may need to be updated or reviewed.

Initial training is essential for a formal PSW role. Several studies have reported the need to identify core peer support competencies in mental health [22,23]. However, there is no consensus on the core training topics required for PSW roles and accreditation [24]. Given the advances in digital peer support and lack of evidence about what topics are important in initial PSW training, there is a need to establish the level of consensus about the content of initial PSW training. A specific knowledge gap relates to the extent to which initial PSW training topics can be delivered on the web. Web-based training may offer benefits, including opportunities to access more prospective PSWs on a larger scale and the reduction of costs as compared with face-to-face training but may not be feasible for training in the relational aspects of the PSW role.

### Aims and Objectives

The aim of this study is to conduct a Delphi consultation technique to establish the level of consensus regarding initial PSW training topics. The objectives were (1) to identify training topics; (2) to identify the extent to which each identified training topic can be delivered on the web; and (3) to assess the degree of consensus that exists with respect to findings from objectives (1) and (2) overall, between PSWs and PSW managers versus PSW researchers and between countries with different resource levels.

## Methods

### Overview

This study was conducted as a part of UPSIDES (Using Peer Support in Developing Empowering Mental Health Services), a 5-year (2018-2022) European Union–funded multinational study that aims to replicate and scale up peer support interventions for people with severe mental illness. Ethics approval was obtained from the University of Nottingham, Faculty of Medicine and Health Sciences Research Ethics Committee (FMHS 377-1908). All participants provided web-based informed consent after reading the participant information sheet available on the web.

### Design

A systematized review was conducted to develop a preliminary list of training topics, followed by a three-round Delphi consultation. Round 1 refined the preliminary list, and rounds 2 and 3 established a consensus.

### Participants

Inclusion criteria were age 18 years or above, able to provide informed consent electronically, and one of the following: publication record relating to peer support work (researcher group) or experience of working as a PSW group or clinical or practical experience in training and/or supervision of peer support (manager group). Participants were not screened for inclusion but self-identified as belonging to one or more of the above groups.

### Procedures

#### Systematized Review

First, we conducted a systematized review of initial PSW training manuals to identify a preliminary list of topics for use in initial PSW training. A systematized review includes elements of a systematic review process while stopping short of being a full systematic review [25]. The inclusion criteria were as follows:

- Training manuals designed specifically for mental health PSWs, that is, designed for people with lived experience of mental health conditions, to prepare them to work in a PSW role with other people with mental health conditions.
- Involved face-to-face training, web-based training, or a combination of both.
- Training with and without accreditation or certification was included, as countries vary in their progress toward an accreditation process.
- Training for both a generic PSW role or for work with a specific mental health subpopulation, for example, dual diagnosis with substance misuse, dementia, and young people.
- Published in the English or Arabic language.

Exclusion criteria were as follows:

- Training manuals not specifically for mental health PSWs.
- Does not include initial PSW training, for example, focus is on stigma related to mental health, human rights, peer leadership, consumer-run organizations, gender identity, and childhood trauma.
- Train the trainer manuals.

Training programs were identified from seven sources:

1. The MEDLINE database was searched using the search strategy shown in Multimedia Appendix 1.
2. Gray literature databases (OpenGrey, New York Academy of Medicine’s Grey Literature Report, TRIP, and Health Quality Ontario) were searched using the phrases: “mental health peer support work training manuals,” “mental health consumer provider training manuals,” “certification training for mental health peer support workers,” and “mental health peer support workers’ core competencies.”
3. Google and Google Scholar were searched using the same phrases mentioned above. The first 100 hits were then screened.
4. Massive open online course platforms (Coursera, edX, FutureLearn, Canvas Network, and Independent) were searched using the massive open online course list website [26], using the same phrases mentioned above.
5. The preliminary findings from a related ongoing systematic review [27].
6. The websites of mental health organizations include the Substance Abuse and Mental Health Services Administration [28], National Mental Health Commission [29], Scottish Recovery Network [30], Mental Health America [31], Mental Health Innovation Network [32], Depression and Bipolar Support Alliance [33], and REdeAmericas [34].
7. The websites of PSW certification, accreditation, and professional bodies include the Missouri Peer Specialist [35], Nevada Certification Board [36], National Association of Peer Supporters [37], and Global Mental Health Peer Network [38].

Searches were conducted in April 2019, and no date restrictions were applied. Endnote software was used to collate the identified manuals. Screening and data extraction were equally divided between AC and NI. Both researchers independently analyzed their allocated manuals and discussed any discrepancies with MS (eg, inclusion and exclusion of manuals). The data abstraction table was populated by extracting data relating to country (specific country and country income level), service setting (eg, community or inpatient), target population (eg, general mental health or dementia), training modality (face-to-face, internet-based, or both), training topics (using terms from source documents), definitions or training goals or learning objectives (where specified), and examples of the training exercises.

Both AC and NI independently conducted thematic analysis [39]. Vote counting for each theme was conducted, and the 2 analysts discussed the discrepancies. AC, NI, and MS then conducted a process of data reduction, which involved comparing the training topics within and across themes and merging and integrating subthemes to generate one coherent coding framework or codebook comprising the training topic name and definition.

#### Delphi Consultation

A three-round Delphi consultation was then conducted. Delphi consultation is a systematic method of determining how much agreement exists on a particular topic based on experts’ opinions [40]. The method involves an iterative and multistage process, comprising multiple rounds of questions designed to combine opinions and assess group consensus.

Compared with the traditional Delphi method, which is delivered via questionnaires through face-to-face meetings, a web-based Delphi offers participants the time to deliberate their responses, thus increasing the validity of results, and requires less resources, for example, time and costs [41]. In addition, given the growth of PSW roles internationally and the wide range of peer support programs that exist, the web-based Delphi was chosen as an appropriate method to answer the research question, as it has the potential to access a diverse and large group of experts engaged in peer support from around the world [42]. Compared with other research designs, the Delphi method can produce rigorous and rich data because of multiple rounds and refinement based on response feedback [43]. Limitations include a low level of reliability of judgment among experts, lack of clear methodological guidelines, and difficulty in assessing the degree of expertise included [44].

Participants for the Delphi consultation were recruited through advertising (1) across networks including the Recovery Research Network [45], UPSIDES consortium, Recovery College Network, and Being Network (Australia); (2) by partner organizations including Nottinghamshire Healthcare National Health Service Foundation Trust, Institute of Mental Health; (3) social media, including Twitter; and (4) snowball sampling. Purposive sampling was used to obtain a balance in role (researcher, PSW, and manager) and income level (high, middle, and low).

In each round, prospective participants were invited via email to complete an anonymous web-based survey using the Jisc survey platform (round 1 and 2) or Google Forms (round 3). Participants were sent 2 reminder emails to complete each round of the survey. All correspondence with the participants occurred via email. An initial pilot test of each round was conducted with 5 nonparticipants, which resulted in minor amendments. Round 1 was distributed over eight screen pages, and rounds 2 and 3 were distributed over four pages. The personal information collected was stored separately from the results’ data on a secure webserver, and participants were allocated identification numbers, for example, P001, which were used to track responses and duplicates. Only the research team had access to personal and research data. No incentive was offered for participation. Participants were able to change their responses using a back button but could not complete the survey without answering all questions. Round 1 began in November 2019, and round 3 was completed in July 2020.

In round 1, after reading the participant information sheet outlining the length of time of the survey, how data are stored, where, and for how long, and the purpose of the study, participants gave informed consent and provided sociodemographic details including if they had any experience of web-based training (about anything). Participants then rated each training topic and associated definition for importance using a 4-point Likert scale (“0=Not important at all,” “1=A bit important,” “2=Quite important,” and “3=Very important”). A free-text response was available to participants, providing the opportunity to suggest additional training topics or changes to the topic name or definition. Qualitative data were analyzed thematically. Two analysts (AC and NI) independently reviewed and synthesized responses using the following framework: proposed topic name, change to language or definition, and additional topics to be covered. Agreement on the topics and definitions arising from the first round was determined by further discussion, refinement, and synthesis with a third analyst (MS). The finalized list of training topics was created based on these responses, including the deletion of topics quantitatively rated as not important, refinement of name or definition, and addition of new topics where relevant.

In round 2, participants rated each topic and associated definition for importance using the same rating scale as in round 1, and also rated the extent to which the topic could be delivered on the web using a 4-point Likert scale (“0=No, it can only be delivered through face-to-face training,” “1=Partially, eg, as blended learning with some aspects delivered online and some face-to-face,” “2=Fully online as a moderated online course, ie, with a peer support worker trainer providing online support and moderation,” and “3=It can be fully delivered online as a standalone course without moderation”).

In round 3, participants were shown their own round 2 ratings and the mean round 2 ratings from other participants in (1) their group (researcher, PSW, and manager or supervisor), (2) the income level of their country setting (high, middle, and low), and (3) overall. Participants were asked to rerate each component for importance and web-based delivery using the same rating scales as in round 2.

### Analysis

For the Delphi consultation, strong consensus was defined as at least 80% of participants in the group with the same rating, and moderate consensus was defined as at least 50% of participants in the group with the same rating. An arbitrary threshold for a high- and moderate-level consensus was implemented.

## Results

### Overview

The systematized review identified 32 training manuals in English, comprising face-to-face and web-based PSW training from 14 different countries (Argentina, Australia, Brazil, Canada, Chile, Germany, Ireland, the Netherlands, Norway, Scotland, Sweden, Uganda, the United Kingdom, and the United States). Training varied in length from 54 hours to 1 year, and the manuals covered a range of PSW training from working with adults, older adults, adolescents, and young people. A total of 502 topics and 348 learning objectives or definitions were extracted. The coding framework synthesized from the training manuals comprised 18 themes and is shown in Multimedia Appendix 2. The coding framework was used as the basis for developing 18 topics and associated definitions, as shown in the first column of Multimedia Appendix 3.

The characteristics of the Delphi consultation participants, including the number of responses for each round, are shown in Table 1.

**Table 1:** Characteristics of the Delphi consultation participants (N=110).

Participant characteristics			Value				
			Round 1 (N=110)		Round 2 (n=89)		Round 3 (n=82)
**Age (years), n (%)**							
	21-29	17 (15.4)		13 (14.6)		11 (13.4)	
	30-39	25 (22.7)		19 (21.3)		21 (25.6)	
	40-49	28 (25.4)		24 (26.9)		22 (26.8)	
	50-59	29 (26.3)		24 (26.9)		18 (21.9)	
	60+	10 (9.0)		9 (10.1)		9 (10.9)	
**Gender, n (%)**							
	Female	79 (71.8)		65 (73)		58 (70.7)	
	Male	29 (26.3)		23 (25.8)		23 (28.0)	
	Other	2 (1.8)		1 (1.1)		1 (1.2)	
**Role, n (%)**							
	PSW^a^	49 (44.5)		38 (42.6)		36 (43.9)	
	Manager	36 (32.7)		29 (32.5)		24 (29.2)	
	Researcher	25 (22.7)		22 (24.7)		22 (26.8)	
**Years of experience in role, n (%)**							
	<2	13 (11.8)		11 (12.3)		11 (13.4)	
	2-3	27 (24.5)		18 (20.2)		17 (20.7)	
	4-6	26 (23.6)		22 (24.7)		20 (24.3)	
	7-9	21 (19.0)		17 (19.1)		15 (18.2)	
	≥10	21 (19.0)		19 (21.3)		17 (20.7)	
**Professional qualification, n (%)**							
	Yes	42 (38.1)		34 (38.2)		30 (36.5)	
	No	68 (61.8)		55 (61.7)		52 (63.4)	
**Lived experience of mental health problems, n (%)**							
	Yes	82 (74.5)		67 (75.2)		62 (75.6)	
	No	28 (25.4)		22 (24.7)		20 (24.3)	
**Experience of initial PSW training, mean (SD)**							
	Scale: 0 (no experience) to 10 (very experienced)	6.4 (2.9)		6.4 (3.0)		6.3 (3.14)	
**Experience of web-based PSW training, n (%)**							
	Yes	25 (22.7)		21 (23.5)		21 (25.6)	
**Experience of web-based training on any topic, n (%)**							
	Yes	88 (80.0)		73 (82.0)		67 (81.7)	

^a^PSW: peer support worker.

### Participant Characteristics

The participants came from 21 countries, including higher-income (Australia: n=33; the United Kingdom: n=22; Canada: n=7; Poland: n=7; Germany: n=4; Ireland: n=4; Switzerland: n=4; Israel: n=3; Norway: n=3; Italy: n=2; the United States: n=2; New Zealand: n=1; Belgium: n=1; Singapore: n=1), middle-income (India: n=4; Tunisia: n=2; Brazil: n=1; Egypt: n=1; Argentina: n=1), and lower-income (Uganda: n=6; Tanzania: n=1) countries.

All participants completed round 1 and rated each of the 18 topics for importance. Round 1 ratings and all proposed changes are shown in Multimedia Appendix 3. Analysis of the round 1 free-text responses (n=68) and mean rating scores resulted in multiple refinements to the round 1 topic names and definitions. Although the mean score for *knowledge of mental health* was ranked low compared with other topics, the free-text responses suggested that the definition should be amended to reflect PSW training needs, and this was changed markedly for round 2. Two topics—*role-specific PSW skills and competencies* and *work skills*—were identified as being relevant in some contexts but not others. These context-specific topics were presented separately in a different format for round 2, along with an explanation for the participants. Three additional topics and definitions were created—*PSW supervision*, *developing a career as a PSW*, and *role-specific PSW skills and competencies*—that were adapted from the *subpopulation and specialized modules* topic.

The revised list of 20 topics and associated definitions were used for round 2, which are shown in the fifth column of Multimedia Appendix 3. Of 110 participants, a total of 89 (80.9%) participants completed round 2. The round 2 ratings of importance are shown in Multimedia Appendix 4 and of web-based delivery are shown in Multimedia Appendix 5. Additional comments were received from round 2 participants about the *role-specific PSW skills and competencies* topic, resulting in minor refinements to the definition of *knowledge of mental health* topic. The final list of topics and definitions, which were used in round 3, is shown in Textbox 1.

Of 110 participants, a total of 82 (74.5%) completed round 3. Of these 82 participants, 76 (93%) had completed round 2. The round 3 ratings of importance, ordered by median rating, are shown in Table 2.

**Final list of topics and definitions for initial peer support worker training.**

Topics always needing coverage

- Introduction to peer support and peer support worker (PSW)
  Presenting the local and international history of peer support, survivor or activist grassroots knowledge, and key information on the context of peer support, PSW, principles, and concept of expertise by experience is essential to formal PSWs
- PSW role focus on recovery
  Teaching about the meaning, stages, and culture of recovery, allowing integration into the PSW’s own experiences and practice. Additionally, teaching leadership, supporting informed choice, and working with service users in difficult times
- Approaches, frameworks, and models used in PSWs
  Familiarizing prospective PSWs with approaches and frameworks underlying which peer support could be practiced. For example, the tree of life, coaching frameworks, strengths-based approach, Intentional Peer Support, and Wellness Recovery Action Planning
- Knowledge of mental health
  Introducing prospective PSWs to different frames of understanding of mental health, including nonmedical models of understanding mental distress (eg, Hearing Voices, Alternatives to Suicide, or Mad Studies) and medical models (eg, diagnosis or interventions), the different types of service setting (eg, inpatient units), and the mental health needs of different populations (eg, age groups, dual diagnosis, or marginalized and minority groups)
- Human rights and disability legislation
  Providing training about the meaning and implications of human rights legislation, including regional or national mental health laws and international legislation such as Convention on the Rights of Persons with Disabilities, to inform values-based PSW practice and skills in working within systems to uphold and protect the rights and social justice for people they work with, for example, through advocacy
- Ethics
  Teaching about PSW values, beliefs, and actions, supporting self-reflection and an understanding about mental health practice and accountability including the importance of boundaries, levels of disclosure, and confidentiality
- Cultural competency
  Practicing PSWs in a way compatible with the cultural needs, values, background, and context of people using services
- PSW skills and competencies
  Providing prospective PSWs with essential competencies needed for formal PSWs through an overview of different PSWs’ job descriptions; teaching the importance of maintaining role integrity and reflecting on the essential qualities and values of PSWs
- Lived experience as an asset
  Highlighting how the experience of mental health problems, alongside other peer experiences such as service use, is a central resource for the PSW role; exploring methods and strategies for using lived experience with service users, including the safe, purposeful, and appropriate use of one’s story to benefit others
- PSW well-being
  Supporting self-reflection and offering strategies for PSWs to promote wellness, recovery, and resilience (eg, teaching PSWs about their workplace rights, self-advocacy, stress management techniques, vicarious trauma, self-care, and how to use reflective practice)
- Communication
  Ensuring prospective PSWs have the fundamental connecting skills (eg, listening skills, use of language, and awareness of verbal and nonverbal cues), which facilitate effective communication with service users in different settings and situations, and helping them develop these skills if necessary
- Trauma-informed peer support practice
  Offering peer support to understand and respond to the trauma of people using services to help them regain or achieve wellness and healing
- Crisis management
  Helping PSWs to understand how to respond collaboratively, supportively, respectfully, and empathically to someone in a crisis
- PSWs working with groups
  Training prospective PSWs on the skills needed to start, facilitate, or cofacilitate a peer support group, in addition to understanding the group processes, dynamics, and coproduction practices and addressing any arising issues
- Workplace aspects of PSWs
  Ensuring PSWs have the skills needed to deal with workplace challenges, including knowledge of support options and training in dealing with work-related pressures, such as working with other professionals with conflicting values, workplace culture, organizational structures, exposure to violence, discrimination, bullying, and managing power dynamics and conflict
- Referral and communication with other services
  Ensuring prospective PSWs know about local services and community resources and about formal communication or referral processes to other services; ensuring PSWs are sensitive to the balance between helpful referrals and supporting self-management or being heard
- PSW supervision
  Introducing PSWs to the purpose, types of, and importance of supervision
- Developing a career as a PSW
  Involves teaching prospective peers about the professionalization of the PSW role, including motivational drivers, career development, training opportunities, and financial management

Context-specific topics

- Role-specific PSW skills and competencies
  Equipping PSWs with role-specific skills (eg, motivational interviewing, solution-focused thinking, family therapy approach, intentional sharing, and understanding cognitive behavioral therapy and mindfulness), understanding of service settings (eg, inpatient units and community teams) and the mental health needs of different populations (eg, age groups, dual diagnosis, homelessness, and marginalized and minority groups)
- Work skills
  Teaching the administrative skills of recording and documenting direct mental health care and incidents and other work-related skills, such as time management

**Table 2:** Delphi Consultation round 3 rating of importance (n=82).

Variables	Total	Participants by role				Participants by income level		
		PSW^a^	Manager	Researcher	High		Middle	Low
Population, n (%)	82 (100)	36 (44)	24 (29)	22 (27)	71 (86)		4 (5)	7 (9)
Lived experience as an asset, median (IQR)^b^	3 (0)^c^	3 (0)^c^	3 (0)^c^	3 (0)^c^	3 (0)^c^		3 (0.25)^d^	3 (0)^c^
Ethics, median (IQR)^b^	3 (0)^c^	3 (0)^c^	3 (0)^c^	3 (0)^c^	3 (0)^c^		3 (0)^c^	3 (0.5)^d^
PSW well-being, median (IQR)^b^	3 (0)^c^	3 (0)^c^	3 (0)^c^	3 (0)^c^	3 (0)^c^		3 (0)^c^	3 (0)^c^
PSW role focus on recovery, median (IQR)^b^	3 (0)^c^	3 (0)^d^	3 (0)^c^	3 (0)^c^	3 (0)^c^		3 (0.25)^d^	3 (0.5)^d^
Communication, median (IQR)^b^	3 (0)^c^	3 (0)^c^	3 (0)^d^	3 (0.75)^d^	3 (0)^c^		3 (0)^c^	3 (1)^d^
Crisis management, median (IQR)^b^	3 (0.75)^d^	3 (1)^d^	3 (0)^c^	3 (1)^d^	3 (0.5)^d^		3 (0.25)^d^	3 (0.5)^d^
Introduction to peer support and PSW, median (IQR)^b^	3 (1)^d^	3 (1)^d^	3 (1)^d^	3 (0)^d^	3 (1)^d^		2.5 (1)^d^	3 (1)^d^
Cultural competency, median (IQR)^b^	3 (1)^d^	3 (1)^d^	3 (1)^d^	3 (1)^d^	3 (1)^d^		2.5 (1)^d^	2 (1)
PSW skills and competencies, median (IQR)^b^	3 (1)^d^	2.5 (1)^d^	3 (0.25)^d^	3 (1)^d^	3 (1)^d^		3 (0.25)^d^	3 (1)^d^
Trauma-informed peer support practice, median (IQR)^b^	3 (1)^d^	3 (1)^d^	3 (0.25)^d^	2 (1)	3 (1)^d^		2 (0.25)^d^	2 (0)^c^
Workplace aspects of PSWs, median (IQR)^b^	3 (1)^d^	3 (1)^d^	3 (1)^d^	2.5 (1)^d^	3 (1)^d^		2.5 (1)^d^	2 (1.5)
PSW supervision, median (IQR)^b^	3 (1)^d^	3 (1)^d^	3 (0)^d^	2 (1)^d^	3 (1)^d^		2 (0.25)^d^	2 (1.5)
PSWs working with groups, median (IQR)^b^	2 (0)^d^	2 (2)	2 (0.25)^d^	2 (0)^d^	2 (0)^d^		2 (0)^c^	2 (1)
Knowledge of mental health, median (IQR)^b^	2 (1)	2.5 (1)^d^	2 (1)^d^	3 (1)^d^	2 (1)		2.5 (1)^d^	2 (1)^d^
Approaches, frameworks, and models used in PSW, median (IQR)^b^	2 (1)^d^	2 (1)^d^	2 (1)^d^	2 (0.75)^d^	2 (1)^d^		2.5 (1)^d^	2 (0)^c^
Human rights and disability legislation, median (IQR)^b^	2 (1)^d^	2 (1)^d^	2 (1)^d^	2 (1)^d^	2 (1)^d^		2.5 (1)^d^	2 (0.5)^d^
Referral and communication with other services, median (IQR)^b^	2 (1)^d^	2 (1.25)	2 (0.25)^d^	2 (0.75)^d^	2 (1)^d^		2.5 (1)^d^	2 (1)
Work skills, median (IQR)^b^	2 (1)^d^	2 (1)^d^	2 (0.25)^d^	2 (1)^d^	2 (0)^d^		3 (0.25)^d^	2 (1)
Developing a career as a PSW, median (IQR)^b^	2 (0.75)^d^	2 (1)	2 (0.25)^d^	2 (0)^d^	2 (0.5)^d^		2 (0.25)^d^	2 (0.5)^d^
Role-specific PSW skills and competencies, median (IQR)^b^	2 (0.75)^d^	2 (1)	2 (0)^d^	2 (0.75)^d^	2 (1)		2.5 (1)^d^	2 (0)^d^

^a^PSW: peer support worker.

^b^Scale 0 (low) to 3 (high).

^c^Strong consensus.

^d^Moderate consensus.

The median rating of importance was “Quite Important” or “Very Important” for all topics. Across all participants, the first five topics in Table 2 reached a strong consensus on importance.

The round 3 ratings for web-based deliverability, ordered by median rating, are shown in Table 3.

The round 3 median ratings for web-based delivery indicated that all topics can be delivered partly or fully on the web with moderation but none without moderation. No topics reached a strong consensus for the mode of training delivery. The range of median responses relating to web-based delivery was smaller for PSWs (1-1.5) than for managers (0-2) and researchers (1-2), indicating that PSWs were more consistent in placing importance on some face-to-face training contact.

**Table 3:** Delphi Consultation round 3 rating of web-based delivery (n=82).

Variables	Total	Participants by role			Participants by income level		
		PSW^a^	Manager	Researcher	High	Middle	Low
Population, n (%)	82 (100)	36 (44)	24 (29)	22 (27)	71 (86)	4 (5)	7 (9)
Human rights and disability legislation, median (IQR)^b^	2 (1)	1.5 (1)	2 (2)	2 (1)	2 (1)	3 (0)^c^	2 (1.5)
Developing a career as a PSW, median (IQR)^b^	2 (1)^d^	1.5 (1)	2 (1)^d^	2 (0)^d^	2 (1)^d^	3 (0.25)^d^	2 (1)
Introduction to peer support and PSW, median (IQR)^b^	2 (1)	1 (1)^d^	2 (1)	2 (1.5)	2 (1)	2.5 (1)^d^	1 (1)^d^
Knowledge of mental health, median (IQR)^b^	2 (1)	1 (1)	1.5 (1)	2 (0.75)^d^	2 (1)	3 (0.25)^d^	2 (1)
Role-specific PSW skills and competencies, median (IQR)^b^	1 (0)^d^	1 (0.25)^d^	1 (0.25)^d^	1 (0.75)^d^	1 (0)^d^	1.5 (1)^d^	1 (0)^c^
Referral and communication with other services, median (IQR)^b^	1 (1)^d^	1 (1)^d^	2 (1)	2 (1)^d^	1 (1)^d^	2 (0.25)^d^	1 (1)^d^
Work skills, median (IQR)^b^	1 (1)	1 (1)^d^	1 (1)	2 (0.75)^d^	1 (1)	2 (0.5)^d^	1 (1)
Approaches, frameworks, and models used in PSW, median (IQR)^b^	1 (1)	1 (1)^d^	1 (1)^d^	2 (1)^d^	1 (1)	2 (0.25)^d^	1 (0)^c^
Workplace aspects of PSWs, median (IQR)^b^	1 (1)^d^	1 (1)^d^	1 (1)^d^	2 (1)^d^	1 (1)^d^	2 (0.25)^d^	1 (1)^d^
PSW supervision, median (IQR)^b^	1 (1)	1 (1)	1 (1)^d^	2 (0.75)^d^	1 (1)	2 (0.25)^d^	2 (1)
PSW well-being, median (IQR)^b^	1 (1)^d^	1 (1)	1 (0.25)^d^	1 (1)^d^	1 (1)^d^	2 (0.5)^d^	1 (1)
PSW role focus on recovery, median (IQR)^b^	1 (1)^d^	1 (0)^d^	1 (1)^d^	1 (1)^d^	1 (1)^d^	2 (0.25)^d^	1 (0.5)^d^
Cultural competency, median (IQR)^b^	1 (1)	1 (1)	1 (1)^d^	1 (1)^d^	1 (1)^d^	2 (0)^c^	1 (1.5)
Ethics, median (IQR)^b^	1 (1)^d^	1 (0.5)^d^	1 (0.25)^d^	1 (1)^d^	1 (0)^d^	2.5 (1)^d^	1 (0.5)^d^
PSW skills and competencies, median (IQR)^b^	1 (1)^d^	1 (0)^d^	1 (1)	1 (1)^d^	1 (1)^d^	1 (0.5)^d^	1 (1)^d^
Trauma-informed peer support practice, median (IQR)^b^	1 (1)	1 (1)^d^	1 (1.25)	1 (1)^d^	1 (1)	2.5 (1.25)^d^	1 (0.5)^d^
PSW working with groups, median (IQR)^b^	1 (1)^d^	1 (1)^d^	1 (2)	1 (0)^d^	1 (1)^d^	2.5 (1.25)^d^	1 (0.5)^d^
Crisis management, median (IQR)^b^	1 (1)	1 (1)^d^	0.5 (1)^d^	1 (0.75)^d^	1 (1)	2 (0.5)^d^	1 (0)^d^
Lived experience as an asset, median (IQR)^b^	1 (1)	1 (1)	0 (1)^d^	1 (1)	1 (1)	1 (0.5)^d^	1 (1)^d^
Communication, median (IQR)^b^	1 (1)	1 (1)	0 (1)^d^	1 (1)^d^	1 (1)	1.5 (1.5)	1 (0)^c^

^a^PSW: peer support worker.

^b^Scale 0 (face-to-face) to 3 (fully via the internet).

^c^Strong consensus.

^d^Moderate consensus**.**

## Discussion

### Principal Findings

In this 21-country study, 20 topics were identified that can be recommended for inclusion in the curriculum of a PSW initial training program. There was a strong consensus about the high importance of five topics: *lived experience as an asset*, *ethics*, *PSW well-being*, *PSW role focus on recovery*, and *communication*. There were no substantial differences between role perspectives (PSW, managers, and researchers) and countries with different resource levels relating to importance. All training topics were identified as being partly or fully deliverable on the web, but none could be provided on the web without moderation. There was no consensus about the right balance between face-to-face and web-based training with moderation, even though PSWs were more consistent in identifying the need for a face-to-face training component.

### Strengths and Limitations

A strength of this study is the number of participants (N=110) from different countries (n=21) and the low attrition (round 1-2: 19%; round 1-3: 25%) compared with other Delphi studies [46]. Another strength is that the Delphi was reported in line with the Checklist for Reporting Results of Internet e-Surveys checklist [47]. This study has several limitations. First, there is a need for more representation from middle- and lower-income countries, which might have allowed between-setting differences to emerge, which was not achieved despite purposive sampling efforts. Second, participation in a web-based consultation may be more difficult for people in environments with poorer internet access and intermittent electricity, which may disproportionately affect PSWs. Third, participants were asked if they had completed web-based training earlier but not specifically web-based PSW training, which could then have been further explored in the analysis. In addition, web-based deliverability was not defined and was based on participant judgment rather than evidence from experience. Fourth, the use of two web-based platforms that may have confused participants and were not specifically designed for Delphi consultations. Alternative Delphi-specific platforms exist, including ExpertLens [48], Mesydel [49], and Delphi2 [50]. Fifth, PSW training manuals available in languages other than English or Arabic might have identified a wider range of training topics.

Finally, a full systematic review was not conducted, and the limitations associated with implementing a systematic review include the following: (1) lack of patient, population, intervention, comparison, and outcomes criteria; (2) included and excluded manuals were not listed; (3) methodological quality assessment and reliability of manuals were not explored; and (4) discrepancies between reviewers were not reported.

### Comparison With Other Work

Achieving an international consensus on topics that are of high importance in PSW training is important for three reasons. First, it offers prospective PSWs a description of the tasks and skills involved in the PSW role, which may inform their decision making about whether to train as a PSW. Second, it provides training providers and organizations with a list of core topics that should be covered in the content of initial PSW training programs in all settings and two additional context-specific topics that may be relevant. Third, it provides an evidence base for developing training curricula and a framework for PSW accreditation. The standardization of PSW training across settings and countries is contentious. On the one hand, an international consortium of peer leaders from 6 continents developed an international charter, which defined peer support and identified key principles and guiding values [51]. In conjunction with this Delphi consultation, a framework is emerging that could underpin the international PSW accreditation process. On the contrary, unintended consequences of institutionalizing the PSW role are emerging, with one qualitative study in the United States concluding that it “has the potential to reduce the very centrality of experiential expertise, reproduce social inequalities, and paradoxically impact stigma” [52].

The identification of training topics relevant to specific contexts reflects cultural and organizational influences on implementation [53]. Identified barriers that may lead to context-specific modifications include the lack of credibility of peer worker roles, professionals’ negative attitudes, tensions with service users, struggles with identity construction, cultural impediments, poor organizational arrangements, and inadequate overarching social and mental health policies [54]. These influences can lead to preplanned modifications implemented during initial PSW training, as well as unplanned extensions to the PSW role [55]. Several studies have found that this role extension can reduce role clarity and integrity, such as by incorporating medical ways of working [56] and creating identity conflict [57]. For example, a 10-site comparative case study across England found that different understandings of professionalism and practice boundaries can erode the distinctiveness of the PSW role [58].

PSW training has evolved in response to organizational needs and more recently the COVID-19 global pandemic. In a recent commentary, barriers to implementing web-based peer support in low- and middle-income countries in the context of COVID-19 were identified [59]. The low-to-moderate consensus about web-based delivery of training found in our study indicates that further work is needed to explore the relative costs and benefits of web-based versus face-to-face training.

All topics were rated as candidates for at least partial web-based delivery, which raises two questions. First, what is the role of web-based moderation? In addition to knowledge and skills development, an important component of PSW training is ensuring that participants have the ability to maintain role integrity in a context where many will have to deal with microaggressions [60]. Similarly, a recent editorial identified specific contested areas relating to the role of PSW in restraint, administration of medication, and lone working in the community [61]. These may all be sensitive issues for PSWs to explore in training, for example, due to personal experiences, which may be more difficult to explore in moderated web-based discussions. Furthermore, individuals considering PSW training may struggle with motivation [62] and the pressure to succeed [63], and role challenges can include overwork and symptom recurrence [64]. There is some evidence that a therapeutic alliance in digital interventions is possible [65], but the extent to which the requisite resilience and motivation for the PSW role can be fostered through web-based training delivery is an important future focus.

Second, does web-based training prepare recipients better to deliver web-based peer support? Relationships are central to PSWs [66], and one impact of COVID-19 is to increase the use of web-based approaches by trained PSWs as an alternative relationship medium. Combining web-based and offline peer support has been shown to be a promising concept, with one qualitative Norwegian study of peer support recipients finding it enabled connectedness and allowed individuals to balance anonymity and openness [67]. Web-based training may help future PSWs to have both technological skills and the confidence to engage with PSW recipients on the web. In middle- and low-income countries, this blend of training delivery could also provide an accessible, wide-reaching, and cost-effective approach to increase the availability of PSW training places. A systematic review identified that the role content of PSWs is often underreported [68]. The topics identified in our study can inform the reporting of both the training program and PSW role components in future PSW evaluations.

### Conclusions

This study developed a list of training topics for the initial PSW training. One use is to inform PSW training manuals, such as the UPSIDES PSW training program [69], which is being evaluated in Germany, India, Israel, Tanzania, and Uganda [70]. The use of an evidence-based training curriculum will increase the effectiveness of programs to prepare individuals for working as PSWs.

## Appendix

Multimedia Appendix 1 Search strategy for the systematized review.

Multimedia Appendix 2 Coding framework developed from the thematic synthesis of peer support worker initial training manuals (n=32).

Multimedia Appendix 3 Changes suggested in round 1 of the Delphi consultation (N=110).

Multimedia Appendix 4 Round 2 Delphi consultation rating of importance (n=89).

Multimedia Appendix 5 Round 2 Delphi consultation rating of web-based delivery (n=89).

## Abbreviations

PSW: peer support worker
UPSIDES: Using Peer Support in Developing Empowering Mental Health Services
