# Supplementary material for: Initial Training for Mental Health Peer Support Workers: Systematized Review and International Delphi Consultation
Source: JMIR Ment Health. 2021 May 27;8(5):e25528. doi: 10.2196/25528 (PMC8193486; doi:10.2196/25528)
Supplement: Multimedia Appendix 1 [file mental_v8i5e25528_app1.docx]

**Multimedia Appendix 1: Search strategy for systematised review**

| 1. Mental health/  2. exp mental health services/  3. exp psychotherapy/  4. exp psychiatry/  5. community mental health centers/  6. hospitals psychiatric/  7. substance abuse treatment centers/  8. exp mental disorders/  9. mentally ill persons/  10. ((mental* or psychiatric) adj (ill* or disorder* or disease* or health* or patient* or treatment or hospital*)).tw.  11. ((chronic* or severe*) adj (mental* or psychiatric)).tw.  12. 1 or 2 or 3 or 4 or 5 or 6 or 7 or 8 or 9 or 10 or11  13 ((consumer* or mental health consumer* or survivor* or people with mental illness) adj2 (provide* or service provider*)).tw.  14. (peer adj (train* or tutor* or work* or provider* or service* or support or companion*)).tw  15. 13 or 14  16. 12 and 15 |
| --- |
