# Supplementary material for: Initial Training for Mental Health Peer Support Workers: Systematized Review and International Delphi Consultation
Source: JMIR Ment Health. 2021 May 27;8(5):e25528. doi: 10.2196/25528 (PMC8193486; doi:10.2196/25528)
Supplement: Multimedia Appendix 2 [file mental_v8i5e25528_app2.docx]

**Multimedia Appendix 2: Coding framework developed from thematic synthesis of PSW initial training manuals (n=32)**

| **Theme name**  Sub-themes |
| --- |
| **1. Introduction to peer support and PSW**  Definition and philosophy of peer support; Context and history of peer support work; Benefits, beliefs and values of PSW; Types and models of peer support; Concept of expert by experience; Principles, purpose and charter of peer support work; Peer support work environment |
| **2. Recovery directed peer support work**  History, philosophy and understanding of recovery; Recovery and Peer support; Stages and process of personal recovery; Recovery Dialogues; How to promote resiliency, hope and empowerment; Promoting wellbeing and advocacy; Creating wellness, recovery and support plans; Promoting leadership and advocacy of peers; Relapse and recovery; Creating recovery culture and program environment that promote recovery |
| **3. Understanding the PSW role**  Characteristics and qualities of the peer support worker; Key roles of peer support workers; Establishing, maintaining and concluding peer support relationship; Overview on different PSW Core competencies; Navigating the mental health system; Peer support work and the workplace; Supporting transitions; Critical thinking and problem solving; Approaches to goal setting |
| **4. Communication**  Types of communication; Core connecting skills; Barriers and facilitators of communication; Safe disclosure and boundary setting; Reflective and active Listening; Conflict management skills |
| **5. Lived experience in PSW**  What is a recovery story; Story telling; Methods of storytelling and why and when it matters; Supporting others to share their personal story; Boundaries of storytelling; Tailoring, reframing and repositioning personal stories |
| **6. Trauma informed peer support practice**  Understanding trauma; Principles of trauma informed care |
| **7. Human rights** **and disability legislation**  Universal declaration of human rights; Diversity and social inclusion (attitudes about diversity and social inclusion); Overview of the national and regional mental health acts and policies |
| **8. Referral and communication with other services**  Making Referrals; Accessing Resources; Connecting with community resources |
| **9. Crisis management**  Risk assessment; Categories of risk; Helping peers to prevent and manage crises; Collaborative risk management |
| **10. Ethics**  Ethical dilemmas; Mandatory Reporting and Duty of care |
| **11. Cultural competency**  Culturally sensitive practice, cultural beliefs and cultural appropriateness; Spirituality |
| **12. Wellbeing and self-care of PSWs**  Looking after yourself (coping with compassion fatigue, recognising workplace burnout, maintaining your wellness, coping with transference feelings, stress reduction, formulating plans of self-coping, resilience, building self-direction, self-determination, self-esteem and self-advocacy); Reflective practice |
| **13. Workplace aspects of PSW**  Working effectively in a multidisciplinary team; Workplace harassment, bullying, power, conflicts and integrity in the workplace; How to participate effectively in meetings; Supervision and mentorship; Peer work relationships; Effective organisational skills |
| **14. Administrative/ effective organisational skills**  Record keeping and collecting and managing information; Documentation, electronic health record training and progress notes |
| **15. PSWs Working with groups**  Basics about groups; Starting, facilitating and sustaining groups; Group processes and problem solving |
| **16.** **Approaches, frameworks and models used in PSW**  Overview of frameworks, models and approaches; Types of models, approaches and frameworks used; Tree of life; Coaching frameworks; Person Centred approaches; Wellness Recovery Action Planning (WRAP); Strengths based approach; Values based practice; Motivational Interviewing |
| **17. Knowledge of mental health**  Mental health disorders and diagnoses; Clinical topics; Understanding mental health treatments; Social determinants of health; Substance use |
| **18.** **Sub population and Specialised modules**  Youth peer support work; Older adult; Specialised modules |
