# Supplementary material for: Initial Training for Mental Health Peer Support Workers: Systematized Review and International Delphi Consultation
Source: JMIR Ment Health. 2021 May 27;8(5):e25528. doi: 10.2196/25528 (PMC8193486; doi:10.2196/25528)
Supplement: Multimedia Appendix 3 [file mental_v8i5e25528_app3.docx]

**Multimedia Appendix 3: Changes suggested in Delphi Consultation round 1 (n=110)**

| **Round 1 Topic and definition** | **Delphi round 1 rating of importance**  Median (IQR) | **Proposed change to language and/or definition** | **Rationale proposed by participants for change** | **Round 2 – Topic and definition** |
| --- | --- | --- | --- | --- |
| **Introduction to peer support and PSW**  Presenting historical and key information on the context of peer support, PSW, principles, and concept of expert by experience is essential to formal PSW | 3 (0) | Change definition to '...concept of expertise by experience...'  The history of peer support, survivor/activist grassroots knowledge e.g. Shery Mead, Patricia Deegan and Judi Chamberlain. |  | **Introduction to peer support and PSW**  Presenting the local and international history of peer support, survivor/activist grassroots knowledge, and key information on the context of peer support, PSW, principles, and concept of expertise by experience is essential to formal PSW |
| **Recovery directed peer support work**  This involves acknowledging and promoting through the practice of PSW the meaning, stages, and culture of recovery. Additionally, teaching skills of creating recovery plans, advocacy, leadership skills and working with service users in difficult times ie. relapse | 3 (0) | Change topic to 'Focus on recovery'  "Creating recovery plans" should not become "the work" of Peer Workers. Teaching Peer Workers how to support informed choice  Avoid use of medicalised terminology i.e. relapse | I think it's important that service users are introduced to tools which have been developed by peers because then Peer Workers can offer them to the people they are supporting  I would personally avoid using in a PSW role, unless the person preferred it | **PSW role focus on recovery**  Teaching about the meaning, stages, and culture of recovery, allowing integration into the PSW’s own experiences and practice. Additionally, teaching leadership, supporting informed choice, and working with service users in difficult times |
| **Approaches, frameworks, and models used in PSW**  Familiarising prospective PSWs with the principles of evidence-based approaches and frameworks underlying which PSW could be practiced e.g. tree of life, coaching frameworks, strengths-based approach, and well-recovery action planning | 2 (1) | The term evidence base is medicalised. Why isn't the hearing voices approach in there? Or IPS?  Well-recovery action planning" - Wellness Recovery Action Planning (WRAP)? | I ranked lower because of the term evidence based, and what was left out | **Approaches, frameworks, and models used in PSW**  Familiarising prospective PSWs with approaches and frameworks underlying which PSW could be practiced. For example, the tree of life, coaching frameworks, strengths-based approach, Intentional Peer Support (IPS) and Wellness Recovery Action Planning (WRAP) |
| **Knowledge of mental health**  Introducing prospective PSWs to different mental health diagnoses, treatment approaches, and interventions | 2 (2) | Q17. Is only important because our lived experience may not have exposed us to the varying experiences of others. But it must be sensitive training that respects diverse views and experiences  Introduce Peer Workers to other frames of understanding, alternative models of understanding distress and other approaches and interventions beyond medication and therapy (e.g. the Hearing Voices approach, Alternatives to Suicide, etc.)  Alternative models of understanding mental distress other than dominant biomedical paradigms e.g. from the Spiritual Crisis Network, Recovery in the Bin etc. | The processes of taking back power, finding our worldview, finding what helps and sticking to it, that's more important  This definition appears to only encompass the medical understanding of mental illness since it talks about "diagnoses"  Alter the wording so that it separates Peer Work from a clinical role and focusses on recovery and strengths-based practice (perhaps presenting these from the LE perspective rather than the diagnostic perspective?) | **Knowledge of mental health**  Introducing prospective PSWs to different frames of understanding of mental health, including non-medical models of understanding mental distress (e.g. Hearing Voices, Alternatives to Suicide, Mad Studies) and medical models (e.g. diagnosis, interventions) |
| **Human rights and disability legislation**  Familiarising prospective PSWs with the universal declaration of human rights in order to practice values-based PSW and an overview of regional and national mental health laws and regulations | 3 (1) | Change definition to 'Providing training about the meaning and implications of human rights legislation, including regional and national mental health laws and regulations, to inform values-based PSW practice'  Peers should understand the philosophy of human rights, the social, mental and emotional implications of rights breaches and restrictions, and pathways for upholding and protecting rights for the people they work with  The implications of local MH acts | I expect peer workers to understand the key messages in the CRPD and CAT as well, plus local laws that protect human rights, and the implications of local MH acts. I have long valued Shery Mead's assertion that peer workers are more like human rights workers than health workers - and so rights are always central to how I have worked, and to how I have supervised and trained other peer workers | **Human rights and disability legislation**  Providing training about the meaning and implications of human rights legislation, including regional / national mental health laws and international legislation such as CRPD, to inform values-based PSW practice and skills in working within systems to uphold and protect the rights and social justice for people they work with, e.g. through advocacy |
| **Ethics**  A complex set of values, beliefs, and actions prospective PSWs need to hold, practice and be held accountable for in mental health practice | 3 (0.75) | Handling confidentiality  Respecting and understanding boundaries including professional and personal boundaries  Levels of disclosure | Both physical and emotional boundaries in keeping oneself and the people we work with safe | **Ethics**  Teaching about PSW values, beliefs, and actions, supporting self-reflection and an understanding about mental health practice and accountability including the importance of boundaries, levels of disclosure, and confidentiality |
| **Cultural-competency**  Practicing PSW in a way compatible with the cultural needs, values, background, and context of peers | 3 (1) | Use another term (e.g. 'people using services') not 'peer' for recipients of PSW |  | **Cultural-competency**  Practicing PSW in a way compatible with the cultural needs, values, background, and context of people using services |
| **Understanding the peer support worker role**  Providing prospective PSWs with essential competencies needed for formal PSW through an overview of different PSWs’ job description, and reflecting on the essential qualities of PSWs | 3 (0) | Add ' maintaining role integrity' to definition  The values that underpin peer support. | Working with other supports and/or carers | **PSW skills and competencies**  Providing prospective PSWs with essential competencies needed for formal PSW through an overview of different PSWs’ job descriptions. Teaching the importance of maintaining role integrity, and reflecting on the essential qualities and values of PSWs |
| **Lived experience in PSW**  Highlighting how the experience of mental health problems is central to a career pathway in PSW. Methods and strategies of using the lived experience with service users will be explored including personal storytelling | 3 (0) | Change topic to 'Lived experience as an asset'  Change definition to 'the experience of mental health problems is a central resource for the PSW role... are explored, including use of one's own story to benefit others'  The safe, purposeful, and appropriate use of personal life and lived experience rather than ‘telling your story’. This is key, not in story telling but as the main source of knowledge.  Relevance of other peer experiences beyond mental health challenges | Service system use and relevant experiences of power & rights infringements are critical. It’s not just about a shared experience of distress. I believe peer workers in clinical settings need to also have lived experience of using a similar service, of losing power and rights | **Lived experience as an asset**  Highlighting how the experience of mental health problems, alongside other peer experiences such as service use, is a central resource for the PSW role. Methods and strategies for using lived experience with service users are explored, including the safe, purposeful, and appropriate use of one’s story to benefit others |
| **Wellbeing and self-care of PSWs**  Strategies prospective PSWs need to learn and practice to promote, maintain wellness and bounce back in case of adversity | 3 (0) | Change topic to 'PSW wellbeing'  Importance of self-recovery  The ability to reflect and process, and how to use this  How to use a reflective practice space  Teaching Peer Workers about their workplace rights such as the right to request reasonable adjustments  Relaxation techniques  Vicarious trauma and self-care | Recognising where you need to be in your journey to be able to work in the peer space  I guess I think the most important tool of all for PSW's is the ability to reflect and process...to release anger and work within an unjust system that has most likely damaged them but in a way that promotes happiness and peace and healing  There are things Peer Workers can do to support their own health and wellbeing but we can't forget that workplaces also have a responsibility to provide a safe workplace and recognise how the workplace may re-traumatise | **PSW wellbeing**  Supporting self-reflection and offering strategies for PSWs to promote wellness, recovery and resilience. For example, teaching PSWs about their workplace rights, self-advocacy, stress management techniques, vicarious trauma, self-care, and how to use reflective practice |
| **Communication**  Equipping potential PSWs with the fundamental connecting skills which facilitate effective communication with service users in different settings and situations | 3 (0) | Emphasise listening skills  Awareness of verbal and non-verbal clues  Add to definition ‘Making sure that …helping them develop these skills if necessary’  Importance of language | I felt my initial training was insufficient in the area of ‘being skills | **Communication**  Ensuring prospective PSWs have the fundamental connecting skills (e.g. listening skills, use of language, awareness of verbal and non-verbal cues) which facilitate effective communication with service users in different settings and situations, and helping them develop these skills if necessary |
| **Trauma-informed peer support practice**  A process of understanding and responding to the trauma of peers to help them regain or achieve wellness and healing through peer support practice | 3 (1) | Change definition to 'Offering peer support to understand and respond to the trauma of people using services, in order to help them regain or achieve wellness and healing' |  | **Trauma-informed peer support practice**  Offering peer support to understand and respond to the trauma of people using services, to help them regain or achieve wellness and healing |
| **Crisis management**  A systematic and collaborative approach of responding to and managing emergency situations to help peers understand categories of risk and how to prevent and manage mental health crises | 3 (1) | ‘Managing’ people or their experiences - this is highly clinical language and inappropriate in a peer space  Peers knowing how to respond supportively, respectfully and empathically to someone in a crisis  De-escalation techniques | Peer support workers should not be involved in risk assessments  Peers should never be i the business of 'managing' people or their experiences - this is highly clinical language and inappropriate in a peer space | **Crisis management**  Helping PSWs to understand how to respond collaboratively, supportively, respectfully, and empathically to someone in a crisis |
| **PSWs working with groups**  Training prospective PSWs on the skills needed to start, facilitate or co-facilitate a peer support group in addition to understanding the group processes and solve any issue happening in the group | 2 (1) | Change definition to 'Training prospective PSWs in the skills needed to facilitate or co-facilitate a peer support group, with some skills in understanding group processes and addressing any arising issues'  Would be improved by adding coproduction practices to group development  Seems that "PSWs working with groups" would be very relevant for initial training of PSWs intending to work in a group setting-- but not relevant to those carrying out individual PSW?  Group Dynamics |  | **PSWs working with groups**  Training prospective PSWs on the skills needed to start, facilitate or co-facilitate a peer support group, in addition to understanding the group processes, dynamics, coproduction practices, and addressing any arising issues |
| **Workplace aspects of PSW**  Teaching PSWs the skills needed to work in multidisciplinary teams and overcome the challenges encountered in the workplace e.g. workplace bullying, power, and conflicts | 3 (1) | Most PSWs I know are highly skilled in their own wellbeing - precisely because of their own journey. Relevance in training PSWs about the kinds of pressures, bullying, discrimination, exposure to violence and more that we so often face in the workplace  Orientation to existing employee support options  Managing conflict  Managing power and mutuality in the peer relationship | This often comes as a shock, and it's worth preparing people | **Workplace aspects of PSWs**  Ensuring PSWs have the skills needed to deal with workplace challenges, including knowledge of support options and training in dealing with work-related pressures, e.g. working with other professionals with conflicting values, workplace culture, organisational structures, exposure to violence, discrimination, bullying, managing power dynamics and conflict |
| **Referral and communication with other services**  Acquainting prospective PSWs with the services and resources in different mental health settings and the community. Additionally, training on formal communication pathways with services e.g. referral processes | 2 (1) | Change definition to 'Ensuring prospective PSWs know about local services and community resources, and about formal communication / referral processes to other services'  Yes and no. Teaching about services and supports has to be done carefully with the caveat that, while we all need assistance at times and we all have a need for human connection, referring someone to a service isn't necessarily the answer (and it certainly isn't an "intervention") | This has to be balanced with teaching Peer Workers to really hear what people's needs are. Services play an important part in a lot of people's lives at one time or another but, as Peer Workers, we also want to be able to support people to draw on their own innate strengths and talents | **Referral and communication with other services**  Ensuring prospective PSWs know about local services and community resources, and about formal communication / referral processes to other services. Ensuring PSWs are sensitive to the balance between helpful referrals and supporting self-management / being heard |
| **Additional topic** |  |  | Regular interchange of emotions and thoughts about first experiences To reflect the own experience and can learn from other's about their experiences  How to actively participate in supervision  Peer Supervision overview - purpose etc. and how it interacts with Line Management Supervision | **PSW supervision**  Introducing PSWs to the purpose, types of, and importance of supervision |
| **Additional topic** |  |  | Links to work experience / traineeship opportunities  Motivational drivers to be PSW. How it can be related to career development?  Income generation identification and management, Financial management as PSW | **Developing a career as a PSW**  This involves teaching prospective peers about the professionalization of the PSW role including motivational drivers, career development, training opportunities, and financial management |
| **Additional topic**  Based on comments from **Subpopulation and specialised modules topic** |  | Nuances of the role in Context Specific Settings (Emergency Rooms, Inpatient Units, ACT Teams, etc.) | Motivational interviewing  Family therapy/approach  Intentional Sharing (IPS Model techniques)  Communication with family members.  Care givers burden.  CBT and mindfulness  Solution focused thinking | **(Context specific) Role-specific PSW skills and competencies**  Equipping PSWs with role-specific skills (e.g. motivational interviewing, solution focused thinking, family therapy approach, intentional sharing, understanding CBT and mindfulness), understanding of service settings (e.g. inpatient units, community teams) and the mental health needs of different populations (e.g. age groups, dual diagnosis, homelessness, marginalised and minority groups) |
| **Clerical/effective organisational skills**  Teaching the non-direct clerical skills of recording and documenting direct mental health care and incidents | 2 (0.75) | Change 'clerical' to administrative'  Peers need to resist practices like writing in the 3rd person, or writing anything the person wouldn't agree with, and be scrupulously transparent and participatory in notes  Time Management | Must be from a peer perspective. | **(Context specific) Work skills**  Teaching the administrative skills of recording and documenting direct mental health care and incidents, and other work-related skills such as time management |
