# Supplementary material for: Initial Training for Mental Health Peer Support Workers: Systematized Review and International Delphi Consultation
Source: JMIR Ment Health. 2021 May 27;8(5):e25528. doi: 10.2196/25528 (PMC8193486; doi:10.2196/25528)
Supplement: Multimedia Appendix 5 [file mental_v8i5e25528_app5.docx]

**Multimedia Appendix 5: Round 2 Delphi Consultation rating of online delivery (n=89)**

| Median (IQR)  [Scale 0 (face-to-face) to 3 (fully online)] | **All** | **Participants by role** | | | **Participants by income level** | | |
| --- | --- | --- | --- | --- | --- | --- | --- |
|  |  | **PSW** | **Manager** | **Researcher** | **High** | **Middle** | **Low** |
| n | 89 | 38 | 29 | 22 | 77 | 6 | 6 |
| Developing a career as a PSW | 2 (1) | 2 (1) | 1 (1) | 2 (1) | 2 (1) | 2 (0.75) | 2 (1.5) |
| Knowledge of mental health | 2 (1) | 1 (1) | 1 (1) | 2 (2) | 2 (1) | 2.5 (1.75) | 1.5 (1) |
| Referral and communication with other services | 2 (1) | 1 (1) | 2 (2) | 2 (1) | 1 (1) | 2 (0.5) | 2 (1.5) |
| Human rights and disability legislation | 2 (2) | 2 (1.75) | 2 (1) | 2 (1) | 2 (1) | 3 (0.75) | 2.5 (1.75) |
| Introduction to peer support and PSW | 2 (2) | 1 (1) | 2 (2) | 2 (2) | 2 (2) | 3 (0.75) | 1 (0.75) |
| PSW role focus on recovery | 1 (1) | 1 (0) | 1 (1) | 1 (1) | 1 (0) | 2 (0) | 1 (0.75) |
| PSW wellbeing | 1 (1) | 1 (1) | 1 (1) | 1 (1) | 1 (1) | 2 (0.75) | 2 (1.5) |
| Crisis management | 1 (1) | 1 (1) | 0 (1) | 1 (1.75) | 0 (1) | 1.5 (1) | 1 (0.75) |
| Approaches, frameworks, and models used in PSW | 1 (1) | 1 (1) | 1 (1) | 2 (2) | 1 (1) |  | 1 (0.75) |
| PSW skills and competencies | 1 (1) | 1 (1) | 1 (2) | 2 (1) | 1 (2) | 2 (0.75) | 2 (0.75) |
| PSW supervision | 1 (1) | 1 (2) | 1 (1) | 2 (1.75) | 1 (1) | 2.5 (1) | 3 (1.5) |
| Workplace aspects of PSWs | 1 (1) | 1 (1) | 1 (1) | 2 (0.75) | 1 (1) | 2 (0) | 1.5 (1) |
| Cultural competency | 1 (1) | 1 (0) | 1 (1) | 1 (1) | 1 (1) | 2 (1.5) | 1 (0) |
| Work skills | 1 (1) | 1 (1) | 1 (1) | 2 (2) | 1 (1) | 2 (1.5) | 1.5 (1) |
| Role-specific PSW skills and competencies | 1 (1) | 1 (1) | 1 (1) | 1 (1) | 1 (1) | 1 (0.75) | 1 (0.75) |
| PSWs working with groups | 1 (1) | 1 (1) | 1 (1) | 1 (2) | 1 (1) | 1.5 (1) | 1 (2.25) |
| Trauma-informed peer support practice | 1 (2) | 1 (1) | 1 (1) | 1 (1) | 1 (1) | 2 (1.5) | 2 (2.25) |
| Ethics | 1 (2) | 1 (1) | 1 (1) | 1 (2) | 1 (1) | 2.5 (1.75) | 2 (0.75) |
| Lived experience as an asset | 0 (1) | 1 (1) | 0 (1) | 1 (1) | 0 (1) | 1 (0.75) | 1 (0.75) |
| Communication | 0 (1) | 0 (1) | 0 (1) | 1 (1.75) | 0 (1) | 1 (0.75) | 1 (1.5) |
